# Functional conservation and divergence of *SEPALLATA*-like genes in floral development in *Cymbidium sinense*

**DOI:** 10.3389/fpls.2023.1209834

**Published:** 2023-08-30

**Authors:** Zeng-Yu Lin, Gen-Fa Zhu, Chu-Qiao Lu, Jie Gao, Jie Li, Qi Xie, Yong-Lu Wei, Jian-Peng Jin, Feng-Lan Wang, Feng-Xi Yang

**Affiliations:** ^1^ Guangdong Key Laboratory of Ornamental Plant Germplasm Innovation and Utilization, Institute of Environmental Horticulture, Guangdong Academy of Agricultural Sciences, Guangzhou, China; ^2^ College of Horticulture and Landscape Architecture, Zhongkai University of Agriculture and Engineering, Guangzhou, China

**Keywords:** *C. sinense*, *SEPALLATA*-like genes, expression pattern, ectopic overexpression, transient expression, protein interaction, floral development

## Abstract

*Cymbidium sinense* is one of the most important traditional Chinese Orchids due to its unique and highly ornamental floral organs. Although the ABCDE model for flower development is well-established in model plant species, the precise roles of these genes in *C. sinense* are not yet fully understood. In this study, four *SEPALLATA*-like genes were isolated and identified from *C. sinense*. *CsSEP1* and *CsSEP3* were grouped into the *AGL9* clade, while *CsSEP2* and *CsSEP4* were included in the *AGL2/3/4* clade. The expression pattern of *CsSEP* genes showed that they were significantly accumulated in reproductive tissues and expressed during flower bud development but only mildly detected or even undetected in vegetative organs. Subcellular localization revealed that *CsSEP1* and *CsSEP4* were localized to the nucleus, while *CsSEP2* and *CsSEP3* were located at the nuclear membrane. Promoter sequence analysis predicted that *CsSEP* genes contained a number of hormone response elements (HREs) and MADS-box binding sites. The early flowering phenotype observed in transgenic Arabidopsis plants expressing four *CsSEP* genes, along with the expression profiles of endogenous genes, such as *SOC1*, *LFY*, *AG*, *FT*, *SEP3* and *TCPs*, in both transgenic Arabidopsis and *C. sinense* protoplasts, suggested that the *CsSEP* genes played a regulatory role in the flowering transition by influencing downstream genes related to flowering. However, only transgenic plants overexpressing *CsSEP3* and *CsSEP4* caused abnormal phenotypes of floral organs, while *CsSEP1* and *CsSEP2* had no effect on floral organs. Protein-protein interaction assays indicated that CsSEPs formed a protein complex with B-class CsAP3-2 and CsSOC1 proteins, affecting downstream genes to regulate floral organs and flowering time. Our findings highlighted both the functional conservation and divergence of *SEPALLATA*-like genes in *C. sinense* floral development. These results provided a valuable foundation for future studies of the molecular network underlying floral development in *C. sinense*.

## 1 Introduction

*Orchidaceae* is one of the largest families of flowering plants, comprising over 27,000 species grouped into approximately 900 genera. Renowned for their distinct floral patterns, fragrances, colors, and leaf art, *Cymbidium* species are highly sought after across the globe, especially in Asia (Roberts and Dixon, 2008; Sun et al., 2021; Yang et al., 2021; Li et al., 2022). *Cymbidium sinense*, one of the eight traditional Chinese Orchids, is a significant ornamental plant and cash crop (Motomura et al., 2010; Zhu et al., 2015; Yang et al., 2021). As an epiphytic plant, it thrives in well-ventilated and cool, moist environments, typically found at low latitudes. The flowering season of *C. sinense* occurs from January to March, coinciding with the Chinese Spring Festival. However, *C. sinense* has a lengthy juvenile phase and requires over three years of cultivation before blooming. The conditions required for flowering include a prolonged photoperiod that triggers spike formation, a period of approximately 6 months of semi-dormancy, and finally a phase of low temperature that promotes inflorescence elongation and flowering (Huang et al., 2012; Yang et al., 2019; Ahmad et al., 2022).

A typical angiosperm flower consists of four whorls of floral organs, namely sepals, petals, stamens, and carpels (Robles and Pelaz, 2005). The ABCDE model (Fan et al., 1997) explains that the combination of A- and E-class genes controls sepal identity, while petal identity is regulated by the protein complexes of A, B, and E-class genes. Similarly, male stamen identity is specified by the expression of B, C, and E-class genes, and female carpel identity by C and E-class genes. Ovule development, on the other hand, is regulated by C, D, and E-class genes (Theissen, 2001; Theissen and Saedler, 2001; Ditta et al., 2004). Given their role in the MADS protein-protein interaction network, SEP-like proteins are essential “hub” proteins (Ruokolainen et al., 2010; Hugouvieux and Zubieta, 2018).

The E-class MADS-box genes, which include *SEP1*, *SEP2*, *SEP3*, and *SEP4*, are essential for determining floral organ identity and are involved in floral meristem identity (Pelaz et al., 2000; Pelaz et al., 2001). The *SEP* genes have undergone multiple duplications, leading to two clades known as *SEP3* (*AGL9*) and *LOFSEP* (*AGL2/3/4* or *SEP1/2/4)*. Subsequent duplications have occurred independently within these clades after the divergence of eudicots and monocots. The *LOFSEP* clades are further divided into *Petunia PhFBP9/23*, *Arabidopsis AtSEP1/2/4* subclades, and *rice SEP* homologous genes *LHS1* and *OsMADS5/34* (Malcomber and Kellogg, 2005; Zahn et al., 2005; Shan et al., 2009). *SEP*-like genes are widely reported in both monocots and eudicots due to their crucial role in regulating floral meristem, floral organs, and flowering period. In eudicots, the *sep1 sep2 sep3* triple mutants and *sep1 sep2 sep3 sep4* quadruple mutants result in sepal-like organs and leaf-like structures in *Arabidopsis*, respectively (Pelaz et al., 2000; Ditta et al., 2004; Castillejo et al., 2005). A mutation in the *fbp2* gene and a double mutation in the *fbp2* and *fbp5* genes in *petunia* cause the transformation of floral organs (Vandenbussche et al., 2003; Matsubara et al., 2008). In monocots, overexpression of the *OsMADS5* gene in rice only promotes early flowering (Jeon et al., 2000), and knockdown of *OsMADS1*, *OsMADS5*, *OsMADS7*, and *OsMADS8* genes simultaneously results in the formation of leaf-like organs (Cui et al., 2010). Additionally, the *SEP* homologous genes have been shown to be crucial for floral organ and floral meristem development in *Gerbera* (Kotilainen et al., 2000), *Lilium* (Tzeng et al., 2003), *maize* (Lid et al., 2004), *Malus* (Ireland et al., 2013), *cucumber* (Wang et al., 2016), *Prunus* (Li et al., 2017), and *tomato* (Zhang et al., 2018), making it an excellent example of duplication and divergence in the evolution of the function of the *SEP* genes in angiosperms (Uimari et al., 2004).

*Orchidaceae* is the largest family of flowering plants and relies on *SEPALLATA*-like genes to maintain floral organ identity (Tsai and Chen, 2006; Salemme et al., 2013). The first E-class gene to be identified in orchids was *OM1*, which was isolated from the *hybrid Aranda Deborah* (Lu et al., 1993). Later, three additional E-class genes (*DOMADS1*, *DOMADS3*, and *DcOSEP1*) were identified in *Dendrobium*, and these genes are sequentially activated during floral transition and persist in mature flowers (Yu and Goh, 2000; Yu et al., 2002). Higher-order complexes resulting from the formation of DcOAP3-DcOPI-DcOSEP1 have been detected via yeast three-hybrid experiments (Xu et al., 2006). Recently, diverse functions of E-class genes have been reported in various orchid species. For example, in *P. equestris*, the *PeSEP3* gene can cause tepals to become leaf-like organs (Pan et al., 2014). Additionally, the expression of *SEP*-like genes has been detected in all floral organs and plays a vital role in the development of the column, lip, and petals in *Habenaria radiata* and *P. henryanum* (Mitoma and Kanno, 2018; Valoroso et al., 2019; Cheng et al., 2022). Defects in *CeSEP1/3*-clade genes also contribute to the leaf-like flower phenotype in *Cymbidium ensifolium* mutants, consistent with research from *Phalaenopsis* (Wei et al., 2020). In contrast, *CeSEP-2* is crucial for the specialized lip and causes the formation of a peloric flower shape in *C. ensifolium* (Ai et al., 2021), unlike in *P. equestris*. Furthermore, the E-class MADS-box *PeMADS8* gene may regulate ovule development and form higher-order complexes with other MADS-boxes (Shen et al., 2021). In *E. pusilla*, *SEP*-like genes *EpMADS8* and *EpMADS9* are involved in orchid fruit development (Lin et al., 2016; Dirks-Mulder et al., 2019).

*C. sinense* is one of the eight traditional Chinese orchid types, valued for its unique floral organ development and flowering time. In this study, we cloned and characterized four *SEPALLATA*-like genes from *C. sinense* and found that these genes exhibited divergent expression patterns. Transgenic Arabidopsis plants expressing all four *CsSEP* genes showed a common phenotype of early flowering, suggesting functional conservation. However, only transgenic plants overexpressing *CsSEP3* and *CsSEP4* displayed abnormal floral organ phenotypes, indicating divergence in *SEPALLATA*-like genes in *C. sinense*. The expression profile of endogenous genes in transgenic Arabidopsis, transient expression in *C. sinense* protoplasts, and protein interaction assays suggested that CsSEP genes could affect downstream-related genes to regulate floral organs and flowering time in *C. sinense*. Our study suggested that *CsSEP* genes played a critical role in regulating floral development in *C. sinense*.

## 2 Materials and methods

### 2.1 Plant materials and growth conditions

The wild-type plants of *C. sinense* ‘Baimo’ utilized in this study were artificially cultivated and collected from the cultivation base of the Environmental Horticulture Research Institute at Guangdong Academy of Agricultural Sciences, China. The plants were grown and maintained in pots inside a greenhouse, with day/night temperatures of 26/23 °C and a 16-h light/8-h dark photoperiod.

### 2.2 Identification and cloning of *CsSEP* genes

Based on the annotated coding sequence (CDS) in the genome database (Yang et al., 2021), we cloned the CDS of *CsSEP1*, *CsSEP2*, *CsSEP3*, and *CsSEP4* using specific primers designed by PrimerPremier 5.0. Total RNA was extracted from natural flowers of *C. sinense* ‘Baimo’ using the RNAprep Pure Plant Kit (TIANGEN, China) following the manufacturer’s instructions. RNA content was measured with a Nano-Drop 2000 Spectrophotometer (Thermo Fisher Scientific, Wilmington, DE), and 1 μg of RNA was used to synthesize first-strand complementary DNA (cDNA) with the HiScript III 1st Strand cDNA Synthesis Kit (Vazyme, China). The cDNA was then used as a template to clone the CDS of *CsSEP* genes with high-fidelity Taq DNA polymerase (Vazyme, China), and the gene-specific primers used are listed in **Supplementary Table S3**. The PCR products were purified using the FastPure^®^ Gel DNA Extraction Mini Kit (Vazyme, China) and cloned into the pCE2 TA/Blunt-Zero Vector (Vazyme, China) for transformation into DH5α (Tiangen, China). Positive clones (8-10) were selected for identification and sequenced by the Sangon Company in Shanghai. The plasmids were extracted with the FastPure^®^ Plasmid Mini Kit (Vazyme, China).

### 2.3 Multiple sequence alignment and phylogenetic analysis

We retrieved partial *SEP*-like genes from previously published studies (Yang et al., 2021; Cheng et al., 2022) and obtained others from the National Center for Biotechnology Information (http://www.ncbi.nlm.nih.gov) for phylogenetic analysis. The registration numbers for these genes are listed in **Supplementary Table S2**. We aligned full-length amino acid sequences using the default settings in ClustalW implemented in MEGA v5.2 (Kumar et al., 2004) and manually adjusted the alignment with the reference alignment provided by Zahn et al. (2005). We used IQ-TREE v1.6.12 (Nguyen et al., 2015) to reconstruct the phylogenetic tree from 1,000 ultrafast bootstrap maximum likelihood (ML) tree replicates. The resulting tree was visualized using Evolview v3 (Subramanian et al., 2019). We performed multiple sequence alignments of *CsSEP* genes against *SEP*-like genes of other known orchids and selected angiosperm species using DNAMAN (v.6.0) software.

### 2.4 Gene expression analysis via qRT-PCR

To verify the spatiotemporal expression patterns of the four *CsSEP* genes, we analyzed different stages of flower buds (S1, S2, S3, S4, S5), root, stem, leaf, flower, pod, sepals, petals, lips, and column (Yang et al., 2021) from *C. sinense* by qRT-PCR. Gene-specific primers were designed for each gene within the non-conservative C-terminal region (**Supplementary Table S3**). qRT-PCR was conducted on a qTOWER 2.0 Real-Time PCR System (Jena, Germany) using ChamQ™ Universal SYBR^®^ qPCR Master Mix (Vazyme) with three biological replications. The qRT-PCR products were amplified in a 20-μL reaction mixture containing 2 μL cDNA, 2 μM of each primer, 10 μL 2×ChamQ Universal SYBR qPCR Master Mix* (Vazyme), and double-distilled water to 20 μL. Briefly, after an initial denaturation step at 95°C for 5 min, the amplifications were carried out with 40 cycles at a melting temperature of 95°C for 15 s, an annealing temperature of 60°C for 30 s, and an extension temperature of 72°C for 30 s, followed by an extra extension step at 72°C for 5 min. In addition, *β-actin* (Mol013347) was selected as an internal reference gene in *C. sinense*. The relative expressions of *CsSEP* genes at the mRNA level were calculated using the 2^−ΔΔCT^ method (Livak and Schmittgen, 2001).

### 2.5 Subcellular localization

The full-length CDS of *CsSEPs* were cloned into the control vector PAN580-GFP using the ClonExpress^®^ II One Step Cloning Kit (Vazyme, China) in accordance with the manufacturer’s instructions. Specific primers with overlapping homologous ends were designed and are listed in **Supplementary Table S3**. The plasmids were extracted using the GoldHi EndoFree Plasmid Maxi Kit (CWBIO, China). The protoplast-based transient expression system (PTES) was used to transform the plasmid DNA into *C. sinense* protoplasts, following the method described by Ren et al. (2020). After incubation for 12-16 h under conditions of 23 °C and darkness, the LSM710 confocal laser scanning microscope was used to visualize the fluorescence of GFP-proteins. To detect cell nuclei, the transfected protoplasts were stained with 50 µg/mL 4’-6’-diamidino-2-phenylindole (DAPI) (Sigma, Germany) at 37 °C for 10 min. DAPI was illuminated by UV light and detected by blue or cyan filters in fluorescence microscopy, and the red self-luminescence of chloroplasts was visualized through the green light of a fluorescence microscope. In addition, three to five images were randomly taken under an LSM710 confocal laser scanning microscope.

### 2.6 Promoter analysis

Using the genome sequence of *C. sinense* as a reference, we selected 2,000 bp of the promoter sequence upstream of the ATG start codon for *CsSEP1*, *CsSEP2*, *CsSEP3*, and *CsSEP4*. We predicted the cis-acting elements and binding sites using the online tools PlantCARE (https://bioinformatics.psb.ugent.be/webtools/plantcare/html/) and PlantPan3.0 (http://plantpan.itps.ncku.edu.tw/promoter.php) and then generated a visual representation using Tbtools.

### 2.7 Ectopic expression of *CsSEP* genes in Arabidopsis

*Arabidopsis thaliana* ecotype Columbia (Col-0) plants were grown under standard glasshouse conditions for use in the functional analysis. We cloned the full-length CDS of *CsSEPs* into the vector pOCA30 using homologous recombination, resulting in four recombinant plasmids: 35S: *CsSEP1*, 35S: *CsSEP2*, 35S: *CsSEP3*, and 35S: *CsSEP4*. These plasmids were introduced into A. tumefaciens GV3101 Chemically Competent Cells (WEIDI, China) by the freeze-thaw method and then transformed into wild-type Arabidopsis using the floral dip method (Clough and Bent, 1998). The seeds collected from the infiltrated plants were sterilized and germinated on 1/2 Murashige & Skoog select medium containing 50 μg/mL kanamycin. We analyzed the expressions of the four *CsSEP* exogenous genes in T2 transgenic plants and wild-type Arabidopsis plants by RT-PCR and normalized the template amounts using the Arabidopsis *Actin2* gene (AT3G18780.2). We evaluated the flowering time in T3 transgenic plants using two reference points. We counted the total number of days required to bolt and the number of rosette leaves at bolting, with no fewer than 10 Arabidopsis plants counted each time.

### 2.8 Expression analysis of endogenous genes in transgenic plants and *C. sinense*

Leaves were harvested from 2-week-old seedlings of 35S: *CsSEPs* transgenic Arabidopsis plants (T2) for total RNA extraction. The expressions of endogenous flowering-related and leaf-related genes, including *AtFT*, *AtSOC1*, *AtSEP3*, *AtTCP20*, *AtGRF2*, *AtGRF1*, *AtTCP3*, *AtARF2*, *AtAG*, and *AtLFY* (Kaufmann et al., 2009; Pajoro et al., 2014), were analyzed by qRT-PCR with three biological replications. The regulations to the downstream genes were verified by overexpressing *CsSEP* genes in *C. sinense* protoplasts. Fluorescein diacetate (FDA) was used to detect the activity of isolated protoplasts under a fluorescent microscope, and those with transfection efficiency exceeding 50% were chosen for subsequent qRT-PCR expression analysis. The regulations to the downstream flowering-related genes *CsSOC1*, *CsFT*, *CsLFY*, *CsAP3-2* and *CsAG1*were analyzed by qRT-PCR with three biological replications.

### 2.9 Yeast two-hybrid assay

The full-length CDSs of *CsSEP1*, *CsSEP2*, *CsSEP3*, *CsSEP4*, *CsAP3-2*, and *CsSOC1* were cloned and recombined into the pGBKT7 and pGADT7 vectors, resulting in the creation of pGBK-CsSEP1, pGBK-CsSEP2, pGBK-CsSEP3, pGBK-CsSEP4, pGAD-CsSAP3-2, and pGAD-CsSOC1, respectively. Each pair of the eight constructs was co-transformed into Y187 and Y2HGold yeast strains, respectively. Diploids containing pGAD-CsAP3-2, pGAD-CsSOC1, and pGBK-CsSEP1/2/3/4 were obtained using a small-scale protocol (Clontech, Palo Alto, USA). After selecting co-transformants in DDO (SD/-Trp/-Leu) medium, they were transferred to QDO/X/A (SD/-Trp/-His/-Trp/-Ade/X-α-gal/AbA) medium. Yeast cells carrying the pGBKT7-53 and pGADT7-T plasmids were used as positive controls, while pGBKT7-Lam and pGADT7-T were co-transformed and served as negative controls. Blue colonies were observed on QDO/X/A plates within 3-5 days, indicating protein interaction between the two proteins.

## 3 Results

### 3.1 Identification and phylogenetic analysis of *CsSEP* genes in *C. sinense*

**Figure 1:**
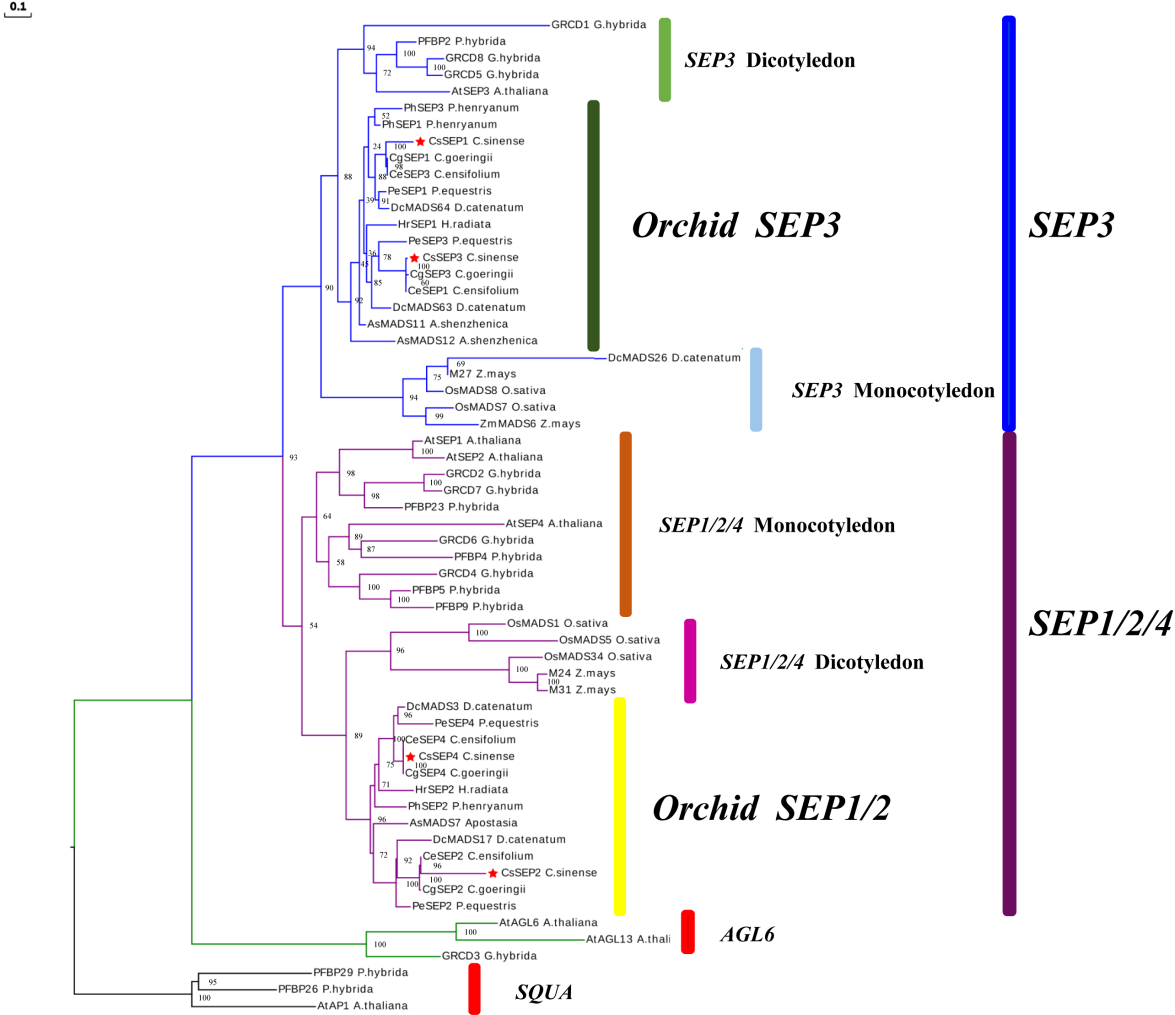
ML-based phylogenetic tree of C. sinense, Phalaenopsis equestris, Cymbidium ensifolium, Paphiopedilum henryanum, Cymbidium goeringii, Apostasia shenzhenica, Dendrobium catenatum, and other selected angiosperm species. The CsSEP1, CsSEP2, CsSEP3, and CsSEP4 genes were marked with red stars.

**Figure 2:**
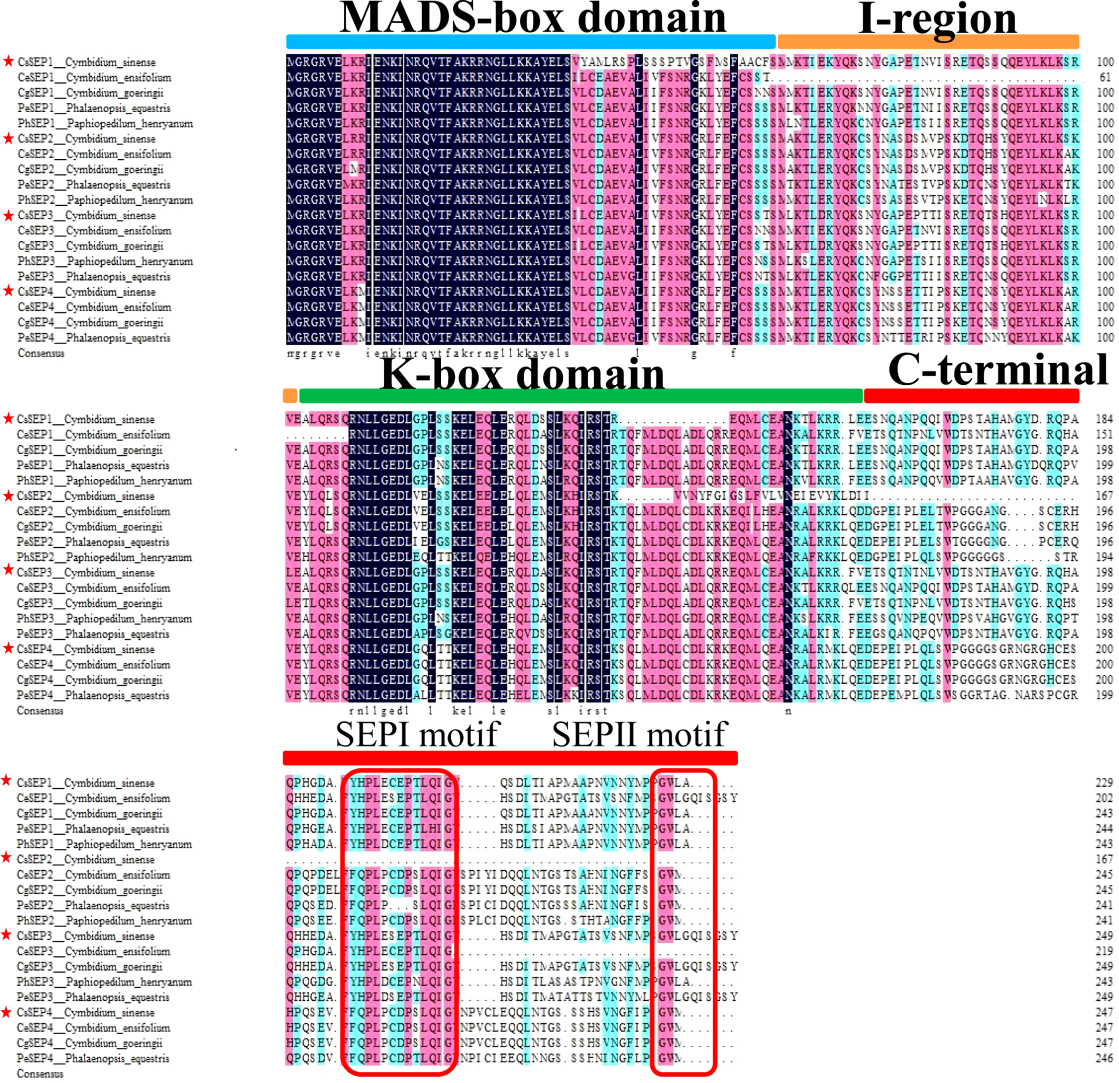
Multiple alignments of the amino acid sequence of *CsSEP1*, *CsSEP2*, *CsSEP3*, and *CsSEP4*. *CsSEP* proteins of *C. sinense* were marked by red stars, which possess the MADS-box domain (light blue line), I-region (orange line), K-box domain (green line), and C-terminal domains (red line). The SEP I and SEP II motifs are indicated with red boxes.

Four *SEP*-like genes are isolated from *C. sinense* ‘Baimo’ and named *CsSEP1*, *CsSEP2*, *CsSEP3*, and *CsSEP4*. Sequence analysis showed that the CDS of these genes contained open read frames (ORFs) of 732, 504, 753, and 744 base pairs, encoding putative proteins of 266, 168, 251, and 248 amino acids, respectively (**Supplementary Figure S1**). The predicted amino acid sequences of these genes shared 34.07% to 60.44% identity (**Supplementary Table S1**). *CsSEP1* and *CsSEP3* showed high homology to *CgSEP1* and *CgSEP3* of *C. goeringii*, with 75.46% and 90.48% identity, respectively. *CsSEP2* shared 52.01% identity with its homolog in *C. ensifolium*, *CeSEP2*. The amino acid sequence of *CsSEP4* was found to be identical to those of *C. ensifolium* and *C. goeringii*, while shared 86.23% identity with *PeSEP4* of *P. equestris*. Based on the amino acid sequences, we constructed a phylogenetic tree to determine the evolutionary relationships of these *SEP*-like genes, which included a number of available orchids *SEP* homologs and other selected angiosperm species (**Supplementary Table S2**). Phylogenetic analysis showed that all the *SEP* genes fell within the *SEP* clade and formed two separate monophyletic clades, with *AGL6* and *SQUA* family members used as outgroups (**Figure 1**). The angiosperm *SEP*-like genes formed two major subclades, the *LOFSEP* clade (also known as the *SEP1/2/4* or *AGL2/3/4* clade) and the *SEP3* (or *AGL9*) clade (Zahn et al., 2005). Based on this analysis, the phylogenetic tree was divided into two major clades (*SEP1/2/4* and *SEP3*), with *CsSEP1* and *CsSEP3* grouped into the orchid *SEP3* (in the *SEP3* clade), and *CsSEP2* and *CsSEP4* included in the orchid *SEP1/2* (in the *SEP1/2/4* clade) (**Figure 1**). Like other monocot lineages, the multiple sequence alignment of the *SEP* genes from orchids showed that *CsSEP* proteins possessed the conserved MIK domain. The C-terminal domain of *CsSEP* proteins had a divergent C-terminal domain with the conserved SEP I motif and SEP II motif (**Figure 2**). Interestingly, both the SEP I motif and SEP II motif of *CsSEP2* were lost together, which is similar to the *SEPALLATA*-like gene *DcMADS26* identified by Zhang et al. (2016) in *D. catenatum* (**Supplementary Figure S2**).

### 3.2 Expression analysis of *CsSEP* genes

**Figure 3:**
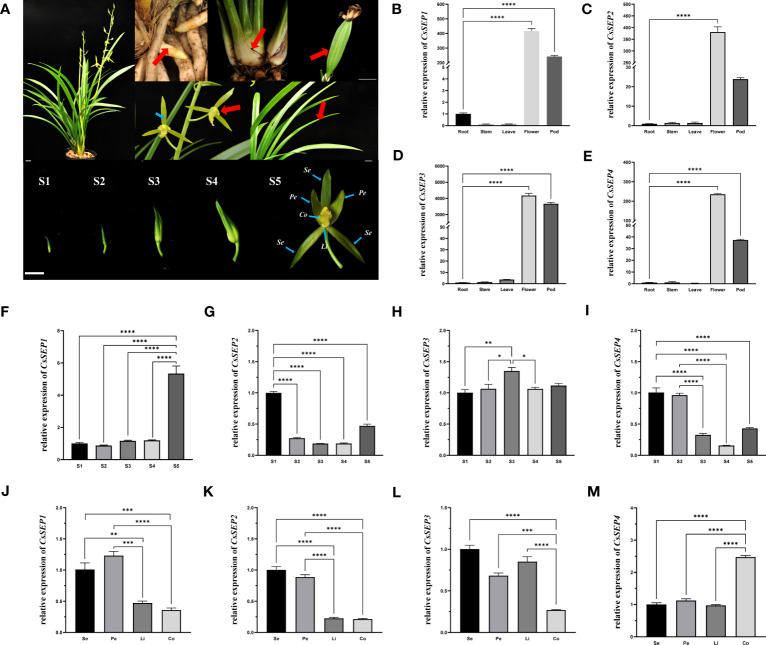
Expression patterns of *CsSEP* genes in *C. sinense*. **(A)** root, stem, leaf, flower, and pod, different stages of flower buds (S1-5) and floral organs in nature *C. sinense* plants. Se: Sepals, Pe: Petals, Li: Lip, Co: Column. **(B–E)** Relative expression patterns of *CsSEP1/2/3/4* in different tissues and organs were examined by qRT-PCR. **(F–I)** Relative expression patterns of *CsSEP1/2/3/4* at five stages of flower buds. **(J–M)** Relative expression patterns of *CsSEP1/2/3/4* in the floral organ. Scale bars: **(A)** 1 cm; Error bars **(B–M)**: ± SD. Significant difference was assessed by One-Way ANOVA and indicated by asterisks; (*)p ≤ 0.05, (**) p ≤ 0.01, (***)p ≤ 0.001, (****) p ≤ 0.0001. Data are expressed as the mean of three biological replicates, with error bars indicating the SD values.

To investigate the role of *CsSEP* genes in floral development, we examined the expression patterns of four *CsSEPs* in various tissues and organs (root, stem, leaf, flower, and pod), as well as floral organs and different stages of developing flower buds (**Figure 3A**). Our findings, as shown in **Figures 3B-E**, indicated that *CsSEP* mRNA were significantly accumulated in reproductive tissues, such as flowers and pods. By contrast, the mRNA of all *CsSEP* genes was only mildly detected or undetected in vegetative organs, such as the root, stem, and leaf. In particular, transcripts of all *CsSEP* genes were highest in flowers, suggesting an essential role in flower development. In terms of the expression pattern of the five flower bud development stages, *CsSEP1*, *CsSEP2*, *CsSEP3*, and *CsSEP4* were expressed throughout all stages of flower bud development (**Figures 3F–I**). Notably, the expression of *CsSEP1* was significantly upregulated in S5 (**Figure 3F**), while transcripts of *CsSEP2* and *CsSEP4* were highest in S1 and gradually downregulated (**Figures 3G, I**). On the other hand, no significant changes were observed in the expression of *CsSEP3* (**Figure 3H**). In the expression profile of floral organs, the expression levels of *CsSEP1* and *CsSEP2* genes in sepals and petals were higher than those in lips and columns (**Figures 3J, K**), while *CsSEP3* mRNA in sepals, petals, and lips was nearly the same, but higher than those in the column (**Figure 3L**). Interestingly, the transcripts of the *CsSEP4* gene were specifically highly expressed only in the column (**Figure 3M**). Based on these results, we concluded that *CsSEP* genes played an indispensable role in floral development, consistent with previous studies, although there were some differences in our findings.

### 3.3 Subcellular localization

**Figure 4:**
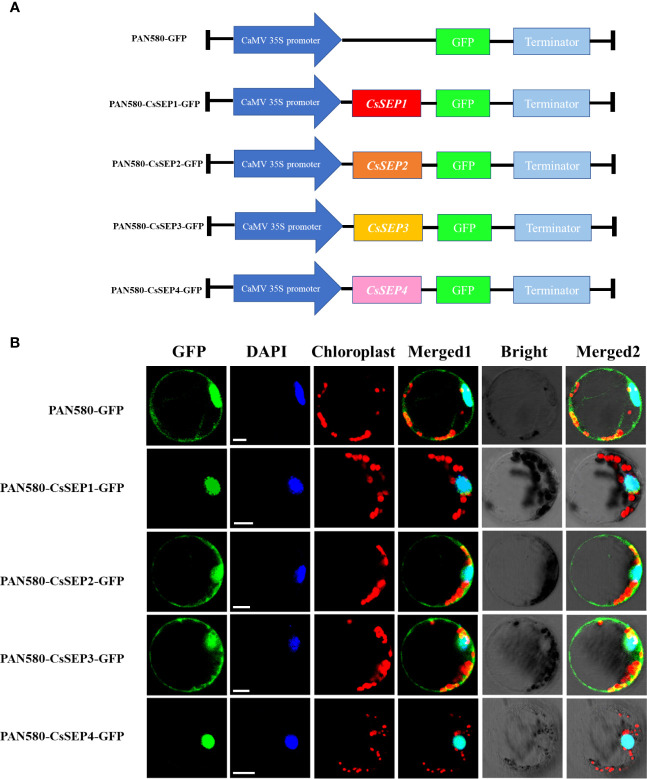
Subcellular localization of *CsSEPs* in *C. sinense* protoplasts. **(A)** Vectors for transient expression were obtained by cloning the full-length CDSs into the vector PAN580-GFP. **(B)** Transient expression of CsSEP fusion protein and the PAN580-GFP control in protoplasts of *C. sinense*, and the fluorescences were visualized through LSM 710 confocal laser microscope. GFP: represents gene localization; DAPI: represents nuclear staining; Chloroplast: represents chloroplast self-luminescence; Scale bars: 10 μm.

To investigate the function of CsSEP proteins, we transformed recombinant vectors expressing fusion proteins PAN580-CsSEP1:GFP, PAN580-CsSEP2:GFP, PAN580-CsSEP3:GFP, PAN580-CsSEP4:GFP, and an empty control vector PAN580-GFP into *C. sinense* protoplasts, using a highly efficient PTES (Ren et al., 2020) (**Figure 4A**). Subcellular localization analysis showed that strong green fluorescence from PAN580-CsSEP1:GFP and PAN580-CsSEP4:GFP was observed in the nucleus, external to the chloroplast signal, and colocalized with the DAPI signal (**Figure 4B**). This finding indicated that CsSEP1 and CsSEP2 were nuclear proteins and consistent with their role as transcription factors (TFs). In contrast, green fluorescence from the fusion proteins PAN580-CsSEP2:GFP and PAN580-CsSEP3:GFP was distributed in both the nucleus and membrane, with the nucleus and chloroplasts being highlighted by blue and spontaneous red light, respectively (**Figure 4B**). Finally, the green fluorescence from the empty control vector PAN580-GFP was distributed throughout the entire cell.

### 3.4 Cis-regulatory elements on the promoters of *CsSEP* genes

**Figure 5:**
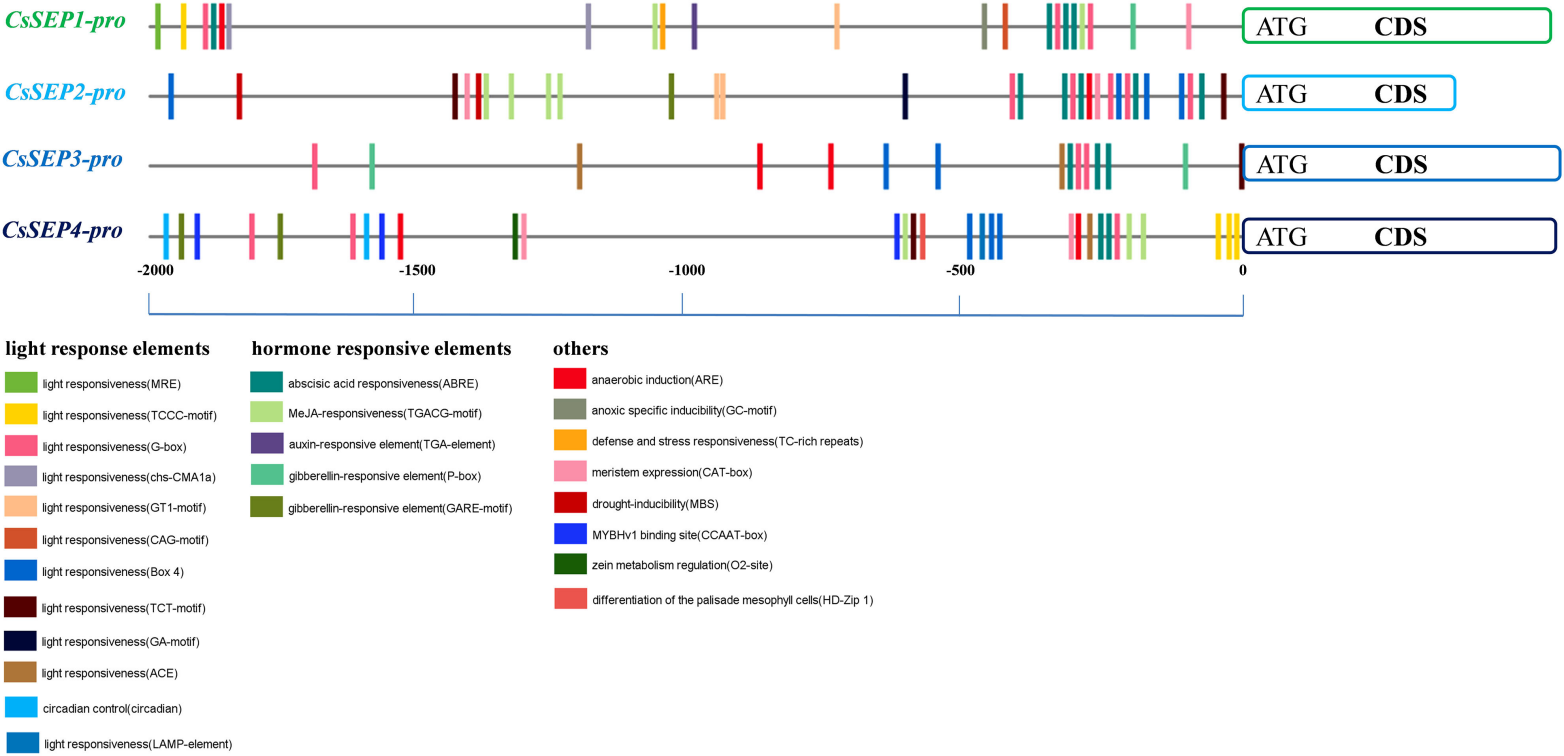
Horizontal lines and short vertical bars of different colors represent promoter regions of *CsSEP1*, *CsSEP2*, *CsSEP3*, and *CsSEP4* genes and the position of putative cis-elements, respectively.

In order to gain a better understanding of the upstream regulation of *CsSEP* genes, we obtained the 2,000 bp promoter region upstream of the coding region based on the genome reference sequence. Using the PlantCARE online website, we predicted the cis-elements in the *CsSEP* promoter region and found that the promoter region contained a large number of core promoter elements. Different types of light response elements (G-box, Box4, GT1-motif, chs-CMA1a, TCT-motif, GA-motif, and TCCC-motif) and HREs (GARE-motif, P-box, TGACG-motif, ABRE, TGA-element, and TCA-element) were widely distributed in the *CsSEP* promoter (**Figure 5**). Notably, the promoter region of *CsSEP4* contained circadian (circadian control), HD-Zip 1 (differentiation of the palisade mesophyll cells), and CCAAT-box (MYBHv1 binding site) motifs. The defense and stress responsiveness element (TC-rich repeats) and auxin-responsive element (TGA-element) were only found in the promoter region of *CsSEP1*, while the drought-inducibility element (MBS) was only present in the promoter region of *CsSEP2*. The meristem expression element (CAT-box) was predicted in the promoter regions of *CsSEP1*, *CsSEP2*, and *CsSEP4* but not in *CsSEP3*. Furthermore, numerous MADS-box transcription factor binding sites were uniformly distributed in the *CsSEP* promoter region predicted from the PlantPan3.0 online website. Specifically, we calculated 16, 22, 12, and 13 MADS-box binding sites in the promoter regions of *CsSEP1*, *CsSEP2*, *CsSEP3*, and *CsSEP4*, respectively (**Supplementary Figure S3**).

### 3.5 Ectopic expression of *CsSEP* genes in Arabidopsis

**Figure 6:**
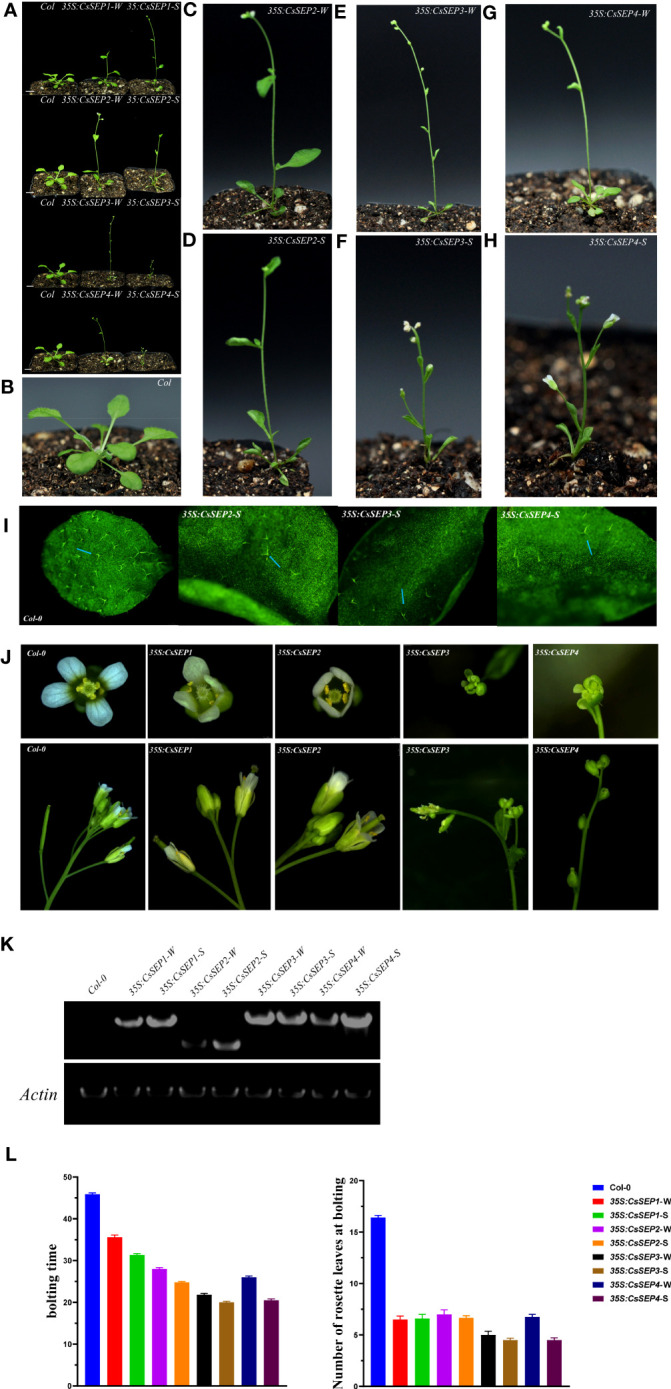
Phenotypic analysis of transgenic *Arabidopsis* ectopically expressing *CsSEP1*, *CsSEP2*, *CsSEP3*, and *CsSEP4* genes. **(A)** Transgenic lines of *35S:CsSEPs* strongly affected the flowering time. **(B)** Wild-type Col-0. **(C-H)** Weak and strong phenotypes of *35S:CsSEP2*, *35S:CsSEP3*, and *35S:CsSEP4* transgenic *Arabidopsis thaliana*, W: weak phenotype transgenic lines; S: strong phenotype transgenic lines. **(I)** Distribution of trichomes in wild-type Col-0 and transgenic *Arabidopsis thaliana* adaxial leaves. **(J)** Abnormal phenotypes of floral organs in *35S:CsSEPs* transgenic *Arabidopsis thaliana*. **(K)** RT-PCR analysis of wild-type Col-0 and *35S:CsSEPs* transgenic *Arabidopsis* lines. An *ACTIN* (*ACT*) gene fragment was amplified as an internal control. **(L)** The number of rosette leaves and bolting time were compared between wild-type Col-0 and *35S:CsSEPs* transgenic *Arabidopsis* plants. Scale bars: 1 cm **(A)**; 0.5 mm **(I)**.

To further investigate the function of *C. sinense CsSEP* genes in flower development, we ectopically expressed *CsSEP1*, *CsSEP2*, *CsSEP3*, and *CsSEP4* genes in Arabidopsis using the cauliflower mosaic virus 35S promoter. After screening transgenic plants, we used three independent, stably overexpressing transgenic lines for further analysis. We found that the four *CsSEP* genes of *C. sinense* produced early flowering in transgenic *A. thaliana*, which could be divided into strong phenotype transgenic lines and weak phenotype transgenic lines based on the curling degree of rosette leaves, flowering time, and plant type (**Figures 6A–H**). The expression levels of the heterologous gene *CsSEP1/2/4* in the strong phenotype transgenic lines (*35S:CsSEP*-S) were higher than those in the weak phenotype transgenic lines (*35S:CsSEP*-W), as detected by RT-PCR (**Figure 6K**).

All four *CsSEPs* transgenic *A. thaliana* showed early flowering under long and short days, the bolting time of Wild-type Col-0 was about 46 days, while the bolting time of *35S:CsSEPs* was about 20 to 35 days. Among them, the earliest bolting time were *35S:CsSEP3*-W and *35S:CsSEP4*-W about 20 days, which were 26 days earlier than wild type Col-0; the next was *35S:CsSEP3*-S about 22 days; and the bolting time of *35S:SEP1*-S was about 35 days, which only 11 days earlier than Wild type Col-0 (**Figure 6L**).

Ectopic expression of *CsSEPs* gene also effected the development of leaves in *A. thaliana*. The number of rosette leaves of wild type Col-0 at bolting were about 17, while the *35S:SEPs* transgenic lines only had 5 to7 rosette leaves (**Figure 6L**). And the different degrees of curled leaves were found in *35S: CsSEP2*, *35S: CsSEP3*, and *35S: CsSEP4*, the degrees of curled leaves in the strong phenotype transgenic lines were more severe than that of weak phenotype transgenic lines, but curled leaves were undetected in the *35S:CsSEP1* transgenic line (**Figures 6A–H**). Additionally, curly leaves on strong phenotypic lines of *35S: CsSEP2*, *35S: CsSEP3*, and *35S: CsSEP4* were exhibited smooth compared with the normal leaves of wild-type Col-0 (**Figures 6B–I**). Microscopic examination revealed that the distribution density of trichomes on the adaxial epidermis of these leaves was much lower than that of wild-type plants (**Figure 6I**). Interestingly, the phenotypes of cauline leaves, terminal flowers and smaller plant size were also found in *35S: SEPs* transgenic lines compared to the wild type Col-0 (**Figures 6A–H**).

*CsSEP* genes not only affected flowering but also played a crucial role in regulating the development of floral organs (**Figure 6J**). In *35S: CsSEP3* had an abnormal stamen and ovule, and the carpel was dehiscent, rendering it unable to develop normal seeds is similar to *Phalaenopsis* (Pan et al., 2014), Particularly, *35S: CsSEP4* transgenic Arabidopsis plants was divergent, the petals transitioned into carpel-like structures, and the floral organs failed to develop in the first and second whorls. However, the phenotypes of *35S: CsSEP1* and *35S: CsSEP2* were consistent with those of wild-type plants, showing no phenotypic changes in floral organ development.

### 3.6 Ectopic expression of *CsSEPs* lead to changes in endogenous genes expressions in Arabidopsis

**Figure 7:**
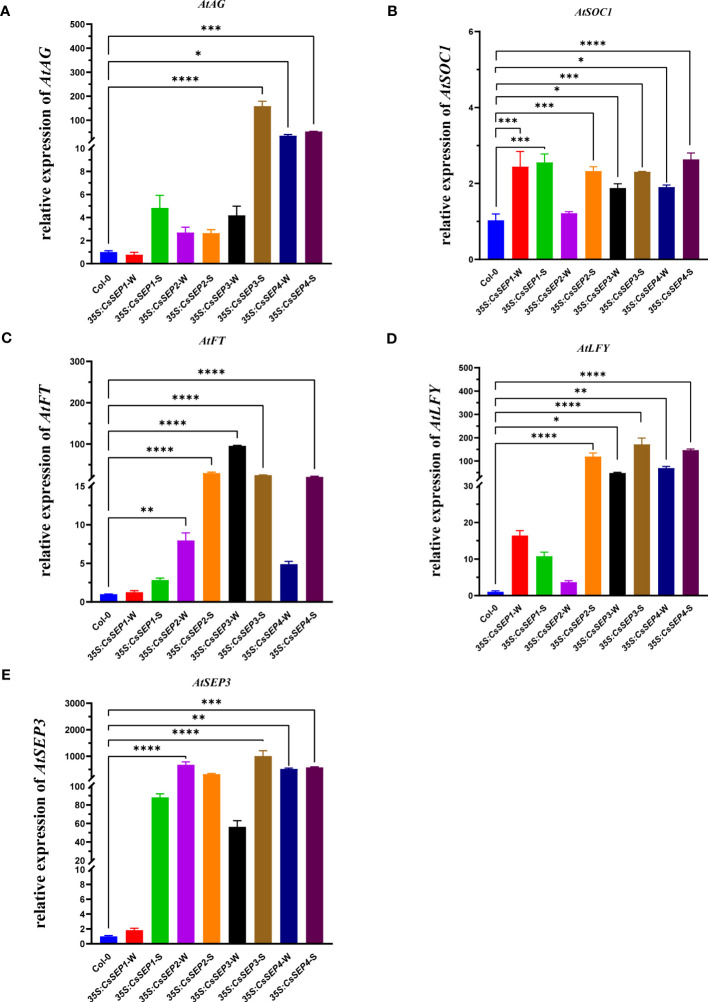
The expressions of **(A)** *AtAG*, **(B)** *AtSOC1*, **(C)** *AtFT*, **(D)** *AtLFY* and **(E)** *AtSEP3* endogenous genes related to floral development were examined with qRT-PCR in wild-type Col-0, *35S:CsSEP1*, *35S:CsSEP2*, *35S:CsSEP3*, and *35S:CsSEP4* transgenic *Arabidopsis*. Significant difference was assessed by One-Way ANOVA and indicated by asterisks; (*)p ≤ 0.05, (**) p ≤ 0.01, (***)p ≤ 0.001, (****) p ≤ 0.0001. Data are expressed as the mean of three biological replicates, with error bars indicating the SD values.

We examined the expression profile of endogenous genes involved in floral and leaf development to elucidate the underlying mechanisms of the observed phenotypes resulting from the ectopic expression of *CsSEPs* in Arabidopsi*s*. Marker genes including *AtFT*, *AtLFY*, *AtSEP3*, *AtSOC1*, *AtAG*, *AtARF2*, *AtGRF1*, *AtGRF2*, *AtTCP3*, and *AtTCP20* were chosen for further analysis. Our results (**Figure 7**) from qRT-PCR assays showed that the expression levels of *AtFT*, *AtLFY*, *AtSEP3*, *AtSOC1*, and *AtAG*, which are related to flowering, were significantly upregulated in *35S:CsSEPs* transgenic plants. The highest increase in mRNA transcription level was observed for *AtSEP3* among these genes, increased by hundreds of times (**Figure 7E**); the second was *AtLFY*, *AtFT* and *AtAG*, increased dozens of times (**Figures 7A, C, D**); the expression of *AtSOC1* also exhibited significant up-regulation, but not as high as *AtFT*, *AtLFY*, *AtSEP3* and *AtAG* (**Figure 7B**). We also examined leaf development-related genes in *35S:CsSEPs* transgenic plants. The transcriptional activation levels of *AtTCP3* and *AtTCP20* in *35S:CsSEP2/34* transgenic lines with curled leaves phenotype were found an upregulated expression, which were not upregulated in *35S:CsSEP1* transgenic lines without curled leaves phenotype (**Supplementary Figures S4A, B**). However, for the transcript levels of *AtARF2*, *AtGRF1* and *AtGRF2*, there were not apparent changed in *35S:CsSEPs* transgenic lines (**Supplementary Figures S4C-E**).

### 3.7 Conserved of *CsSEP*s on regulate floral development genes in *C. sinense in vivo*

**Figure 8:**
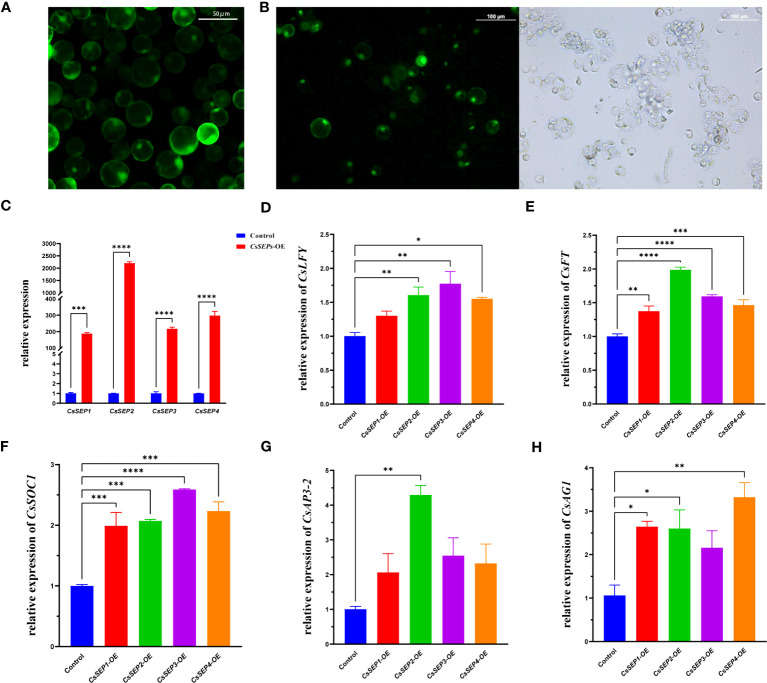
Regulation analysis of *CsSEP1*, *CsSEP2*, *CsSEP3*, and *CsSEP4* proteins to express floral development related genes with qRT-PCR. **(A)** Isolated highly active protoplasts; **(B)** The transfection efficiency of protoplasts; **(C)** The expression level of *CsSEP* genes in *C. sinense* protoplasts, **(D-H)** The expressions of *CsAP3-2*, *CsAG1*, *CsFT*, *CsSOC1*, and *CsLFY* endogenous flower development genes were examined with qRT-PCR in *C. sinense*. Significant difference was assessed by One-Way ANOVA and indicated by asterisks; (*)p ≤ 0.05, (**) p ≤ 0.01, (***)p ≤ 0.001, (****) p ≤ 0.0001. Data are expressed as the mean of three biological replicates, with error bars indicating the SD values.

To gain a deeper understanding of the regulatory mechanism of *CsSEPs in vivo*, we transiently overexpressed *CsSEPs* genes in *C. sinense* protoplasts using a highly efficient PTES. We were able to obtain intact and clean protoplasts with an activity of over 95%, which could be used for further transfection (**Figure 8A**). We successfully transfected the recombinant plasmids into *C. sinense* protoplasts, as detected by fluorescence microscope (**Figure 8B**), and examined the expression of flowering-related genes using qRT-PCR. As expected, the expression levels of *CsSEP1*, *CsSEP2*, *CsSEP3*, and *CsSEP4* were significantly elevated after transfection (**Figure 8C**). We also detected the transcriptional levels of endogenous floral development related genes *CsSOC1*, *CsFT*, *CsLFY*, *CsAP3-2* and *CsAG1* in *C. sinense* protoplasts and found that the expression of *CsSOC1*, *CsAP3-2* and *CsAG1* were significantly upregulated after transfection (**Figures 8F–H**). Moreover, the other two flowering-related genes also showed a trend of up-regulation (**Figures 8D, E**). These results suggested that *CsSEP* genes might regulate floral development by affecting the expression of downstream related genes in *C. sinense*.

### 3.8 *CsSEP* proteins interact with other MADS-box proteins

**Figure 9:**
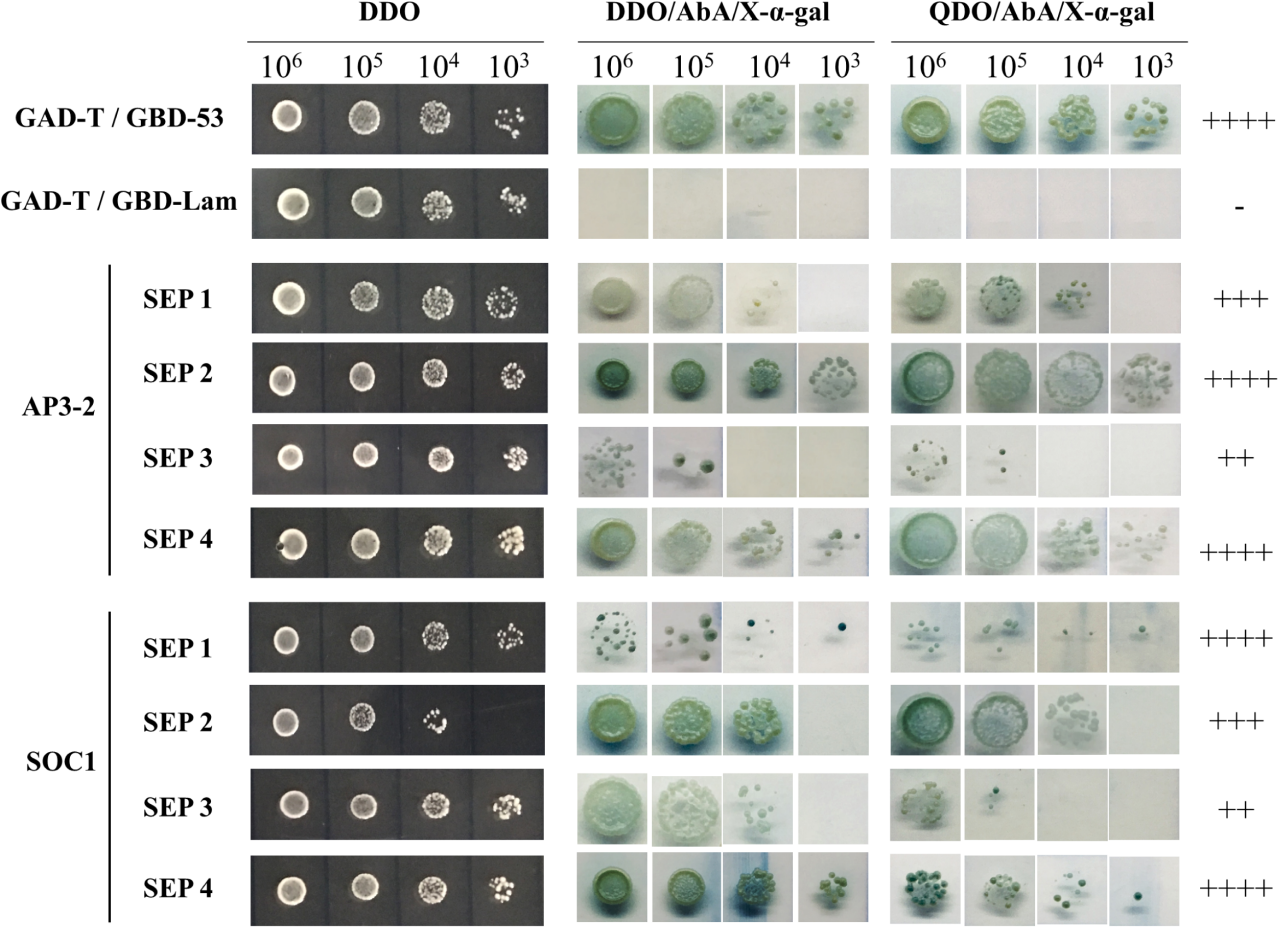
Analysis of protein–protein interactions between CsAP3-2, CsSOC1, and CsSEP1/2/3/4 of *C. sinense* by the yeast two-hybrid system. ‘++++’, ‘+++’, ‘++’ and ‘+’ are very strong, strong, moderate and weak interactions, respectively, on selective plates. ‘-’, no interaction.

In our study, we observed that overexpression of the *CsSEP* genes in Arabidopsis resulted in widespread early flowering and floral organ transformation. To analyze the relationship between CsSEP proteins and other MADS-box proteins, we conducted yeast two-hybrid assays (**Figure 9**). Specifically, we investigated the protein-protein interactions within E-class SEPALLATA-like proteins, B-class protein AP3-2, and MADS-box flowering-related protein SOC1 in *C. sinense*. Our results indicated that yeast cells cotransformed with recombinant vectors pGBK-CsSEP1/2/3/4+pGAD-CsAP3-2 and pGBK-CsSEP1/2/3/4+pGAD-CsSOC1 yielded blue colonies on QDO/X/A medium, consistent with the positive control. This finding suggested that CsSEP1, CsSEP2, CsSEP3, and CsSEP4 showed interactions with CsAP3-2 and CsSOC1 at the protein level. Moreover, the intensity of interaction among these MADS-box proteins varied. Specifically, CsSEP4 showed the strongest interaction with CsAP3-2 and CsSOC1, while the interaction between CsAP3-2, CsSOC1, and CsSEP2 was also very strong. The intensity of interaction between CsSEP1 and CsAP3-2 and between CsSEP2 and CsSOC1 was strong. However, the interaction ability of the CsSEP3 protein showed only moderate intensity to CsAP3-2 and CsSOC1 proteins.

## 4 Discussion

In previous studies of model plants including Arabidopsis (Coen and Meyerowitz, 1991) and *Oryza sativa* (Cui et al., 2010), four *SEP* genes exhibit redundant functions in determining floral development and meristem. In this study, we cloned and identified four *SEP* genes in *C. sinense*. Phylogenetic analysis revealed that *CsSEP2* and *CsSEP4* belonged to the *SEP1/2/4* clade, forming an Orchid *SEP1/2* clade with *C. ensifolium*, *C. goeringii*, and other Orchids, while *CsSEP1* and *CsSEP3* belonged to the *SEP3* clade (**Figure 1**). This finding was consistent with previous reports on the phylogeny of *SEP1/*3-like genes in *P. henryanum* and *P. equestris*. In Orchids, four clades of *SE*P genes have been generated from orchid-specific duplications within monocot *SEP* clades (Pan et al., 2014; Ai et al., 2021; Sun et al., 2021; Yang et al., 2021; Cheng et al., 2022). Therefore, we speculated that the endemic *Orchid SEP1/2* and *Orchid SEP3* branches were formed during plant evolution. The predicted amino acid sequences of *CsSEP* proteins exhibited a high degree of identity with homologous proteins from *P. equestris*, *C. goeringii*, and *C. ensifolium*. As expected, CsSEP proteins possessed the conserved MIK domain, and the C-terminal domain of CsSEP proteins had a divergent C-terminal domain with the conserved SEP I motif and SEP II motif, supporting their characterization as E-class floral meristem identity genes and suggesting a similar functionality (**Figure 2**). However, both the SEP I motif and SEP II motif of *CsSEP2* were lost together, similar to the *SEPALLATA*-like gene *DcMADS26* identified by Zhang et al. (2016) in *D. catenatum* (**Supplementary Figure S2**). This may be due to the loss of *SEP* genes caused by frequent replication during plant evolution and leading to functional divergence. Hence, the extensive duplication of MADS-box genes and the resulting subfunctional and expressional differentiation were associated with the regulation of species-specific flower traits (Elomaa et al., 2018).

In eudicots, *SEPALLATA* homologous genes are found to accumulate to high levels in flower buds while being low in shoot tips and undetectable in leaves and roots (Ampomah-Dwamena et al., 2002; Ma et al., 2022; Zhang et al., 2022). In this study, the expression pattern of *CsSEP* genes in reproductive organs was also found to be conserved. Specifically, the mRNA of *CsSEPs* showed significant accumulation in flowers and pods, but was only mildly detected or even undetected in roots, stems, and leaves (**Figures 3B–E**). These results could be verified in other orchids, such as *C. goeringii*, *E. pusilla*, and *P. henryanum*, where the same expression pattern of *SEP* homologous genes is found to be expressed in floral organs, with low or no expression in vegetative organs (Lin et al., 2016; Dirks-Mulder et al., 2019; Sun et al., 2021; Cheng et al., 2022). However, in the flower bud development process (**Figures 3F–I**), the expression of *CsSEP1* was significantly upregulated in S5. In contrast, the transcripts of *CsSEP2* and *CsSEP4* were highest in S1. No significant change was observed in the expression of *CsSEP3* during the flower bud development stages. These results showed non-overlapping expression profiles temporally. Similarly, the expression levels of *SEP* homologous genes are found to be very different at different floral bud developmental stages (Xiang et al., 2020). Regarding the expression profile of floral organs (**Figures 3J–M**), *CsSEP1* and *CsSEP2* genes in sepals and petals were expressed more highly than those in lips and columns. The *CsSEP4* gene was specifically highly expressed in the column, implying that it played a role in determining the column. In contrast, in *Phalaenopsis*, *PeSEP2* is highly expressed in the column, *PeSEP3* expression is dominant in the petal, and *PeSEP4* expression is extremely low in floral organs (Pan et al., 2014). *SEP3* homologous and *SEP4* homologous genes are mainly highly expressed in the outer two whorls of *marigold* and *gerbera* (Zhang T. et al., 2017; Zhang et al., 2021). The non-ubiquitous expression patterns of *SEPALLATA*-like genes offered a rare opportunity to investigate their functional conservation and divergence in *C. sinense*.

In both model and non-model plants, including *Arabidopsis*, *rice*, *tomato*, *Gerbera* and *Prunus*, *SEP*-like genes are well-conserved floral organ identity genes and key regulators of flower development (Ferrario et al., 2003; Teo et al., 2014; Zhang T. et al., 2017; Zhou et al., 2017). Overexpression of *SEPALLATA*-like genes in Arabidopsis, which is commonly observed, leads to early flowering (Ditta et al., 2004), and this phenomenon is well-conserved in distant angiosperm species. In this study, the four *CsSEP* genes of *C. sinense* produced early flowering in transgenic *A. thaliana* (**Figure 6A**). However, additional phenotypes including smooth curled leaves, reduced plant size and terminal flowers (**Figures 6B–H**) were also observed. However, only one or more *SEP* homologous in other species can cause early flowering phenotype, such as in *Phalaenopsis*, only *PeSEP3* gene can lead to early flowering phenotype in Arabidopsis (Pan et al., 2014), differently, four *CsSEP* genes all showed early flowering in transgenic Arabidopsis, indicating that the functions of *SEPs* genes are not only conserved but also divergent in *C. sinense*.

Considering that the *SEP* homologous genes in different angiosperms are diverse, leading to the homeotic conversion of floral organs and even affecting fruit development (Zhang S. et al., 2017; Qi et al., 2020; Zhang et al., 2021; Zhang et al., 2022). For example, *SEP* homologous genes in *Isatis indigotica*, *Marigold*, and *Gerbera* can affect the structure of sepals, petals, stamens and carpels (Uimari et al., 2004; Ma et al., 2019; Zhang et al., 2021). Additionally, *SEP* homologous genes can effect fruit and seed also have reported in *grapevine* (Zhang et al., 2022). It is reasonable that these homologous genes showed separation of phenotypic traits. In orchids, *C. ensifolium CeSEP2* gene can affect floral organ development (Ai et al., 2021), and the function of *Phalaenopsis PeSEP3* gene affecting the floral organ structure is consistent with the *CsSEP3* gene in this study (Pan et al., 2014), which exhibited an abnormal stamen and ovule, with the carpel being dehiscent and unable to develop normal seeds (**Figure 6J**). These conclusions further reveal that the effection of *SEP* gene on floral organs was conserved. Conversely, the effection of four *CsSEPs* genes on floral organs were divergent from each other in *C. sinense*. In this study, the overexpression of *CsSEP3* and *CsSEP4* caused morphological changes of floral organs, however, the *35S:CsSEP1* and *35S:CsSEP2* showed no phenotypic changes in floral organ development (**Figure 6J**). In particular, *35S:CsSEP4* transgenic plants resulted in the transformation of petals into carpel-like structures, with floral organs failing to develop in the first and second whorls, which has not been reported before. In species that have undergone recent evolution, the impact of *SEP* homologs on flower organs appears to be divergent in function. While the relationship between the four *SEP* genes remains unclear, several studies have suggested that the functions of *SEP1* and *SEP2* are more similar to those of *SEP4*. On the other hand, other reports have supported that the function of *SEP3* is more similar to that of *SEP1* and *SEP2* (Zhang et al., 2021).

The phenotypic changes in transgenic plants were verified to be related to the expression levels of their endogenous genes regulated by *SEP* genes. For instance, in *marigold* and *Platanus acerifolia*, ectopic expression of *SEP* homologous genes regulates the expression of endogenous genes in *A. thaliana* (Zhang S. et al., 2017; Zhang et al., 2021). Consistent with those conclusions, the expression levels of endogenous floral development genes, such as *AtFT*, *AtLFY*, *AtSEP3*, *AtSOC1* and *AtAG*, were significantly upregulated in *35S: CsSEP* transgenic plants (**Figure 7**), and the phenotypic differences might be due to the different capabilities of activating downstream target genes in Arabidopsis. In Arabidopsis, *FT* and *SOC1* were the main flowering signal pathway integrators. *SOC1* can, together with *AGL24*, directly activate *LFY* (Parcy, 2005; Lee and Lee, 2010). These homologous genes in orchids also play a conserved role in influencing flowering. For instance, overexpression of *DOFT* in *Dendrobium* results in increased transcript levels of *DOSOC1* (Wang et al., 2017). Moreover, when the *CsSEP* genes were transiently overexpressed in *C. sinense* protoplasts, the expression levels of *CsSOC1*, *CsFT*, *CsLFY*, *CsAP3-2* and *CsAG1* were shown to have an upregulated trend (**Figures 8D–H**), respectively, which was consistent with the promotive effect of *CsSEPs* on endogenous floral development genes in Arabidopsis. These results indicated that activation of floral development genes by *SEPALLATA*-like genes was well-conserved in *C. sinense*.

SEP proteins act as “glue,” facilitating interactions within MADS proteins and driving the formation of distinct tetrameric complexes (Honma and Goto, 2001; Ditta et al., 2004; Zahn et al., 2005; Immink et al., 2009). In Arabidopsis, B-class genes are direct targets of SEP proteins, and SEP3 displays extensive interactions with other MADS-box proteins (de Folter et al., 2005). Similarly, in orchids, SEP-like proteins bind to CArG-box sequences and interact with AP1/FUL, B, C, D, and other MADS-box proteins during flower development (Tilly et al., 1998; Pan et al., 2014; Mitoma and Kanno, 2018; Cheng et al., 2022). In the evolution of the orchid family, B-class MADS-box genes is diverged into different subclades, and involved with E-class *SEP* genes in the development of a highly modified perianth (Pan et al., 2011; Lucibelli et al., 2021). For example, the loss of class B and E genes leads to the adaxial petal not differentiating into a specialized lip in *Apostasia shenzhenica* (Zhang G. Q. et al., 2017); in *Phalaenopsis*, *PeSEP1* gene plays a role in the control of floral morphogenesis directly or indirectly via interact with two *AP3* homologous genes *PeMADS2* and *PeMADS4* (Pan et al., 2014). Similarly in *C. sinense*, E-class genes were still conserved interact with B-class gene, that was consistent with *Phalaenopsis*. However, different from *Phalaenopsis*, the CsSEP1, CsSEP2, CsSEP3 and CsSEP4 proteins all had different degrees of interaction with only one B-class CsAP3-2 protein (**Figure 9**), this finding further supported the conservative and divergent role of class B and E proteins in regulating floral development in *C. sinense*. Furthermore, protein interaction analysis also detected that CsSEP1, CsSEP2, CsSEP*3*, and CsSEP4 interacted with CsSOC1. Previous studies only confirmed that *SE*P genes can interact with ABCDE-class genes and have not reported interactions with *SOC1*. This new discovery shows that the *CsSEPs* genes had produce functional divergence and may recruit CsSOC1 protein to regulate the flowering transition in *C. sinense*.

## 5 Conclusion

In this study, we isolated and identified four *SEPALLATA*-like genes from *C. sinense* and conducted a systematic analysis of their phylogenetic relationships, protein sequences, and expression patterns. Our findings revealed that *CsSEP* genes were both conserved and divergent in floral initiation and development. Based on our analysis of transgenic phenotypes in Arabidopsis, expression profiles of endogenous genes, and protein interaction patterns, we suggested that *CsSEPs* genes played an important role in regulating flowering time and floral organ development in *C. sinense*. The results of our study on the early flowering phenotype of transgenic plants overexpressing *CsSEP* genes demonstrated their conservative functions in regulating flowering transition. However, the regulation of floral organ development by *CsSEPs* was divergent, suggesting that *CsSEP3* and *CsSEP4* could be potential candidate genes for further research. Furthermore, our study on the different interaction patterns of CsSEP proteins with MADS-box genes shed new light on the conservative and divergent functions of these proteins. We predicted that CsSEPs might form a protein complex with B-class and CsSOC1 proteins to affect downstream genes and regulate floral development. Although the study on the mechanism of orchid flower development is still in its preliminary stage, our findings on the role of *SEPALLATA* genes in regulating floral development in *C. sinense* will lay a foundation for further study of the molecular network.

## Data availability statement

The original contributions presented in the study are included in the article/**Supplementary Material**. Further inquiries can be directed to the corresponding authors.

## Author contributions

F-XY, G-FZ, and F-LW designed the experiments and edited the manuscript, Z-YL, C-QL, and JG executed the experiments and assembled the figures, Y-LW, J-PJ, JL and QX conducted the qRT-PCR, F-XY, F-LW, and Z-YL wrote the paper with inputs from other authors. All authors read and approved the final manuscript.
