# Supplementary material for: Functional conservation and divergence of SEPALLATA-like genes in floral development in Cymbidium sinense
Source: Front Plant Sci. 2023 Aug 30;14:1209834. doi: 10.3389/fpls.2023.1209834 (PMC10498475; doi:10.3389/fpls.2023.1209834)
Supplement: Supplementary file 1 [file DataSheet_1.docx]

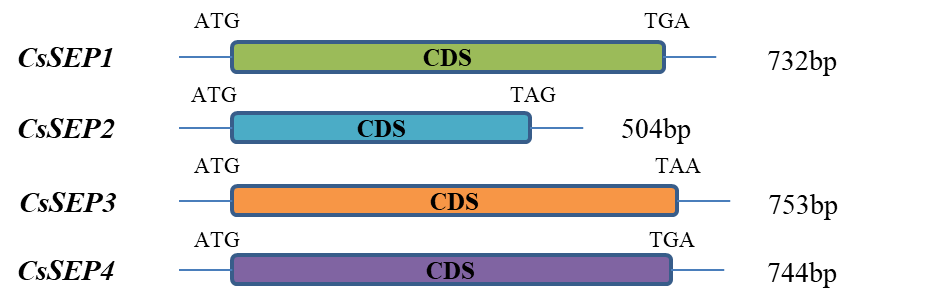


**Supplementary Figure S1**. Schematic diagram of the identified and cloned *CsSEP1*, *CsSEP2*, *CsSEP3*, and *CsSEP4* gene sequences


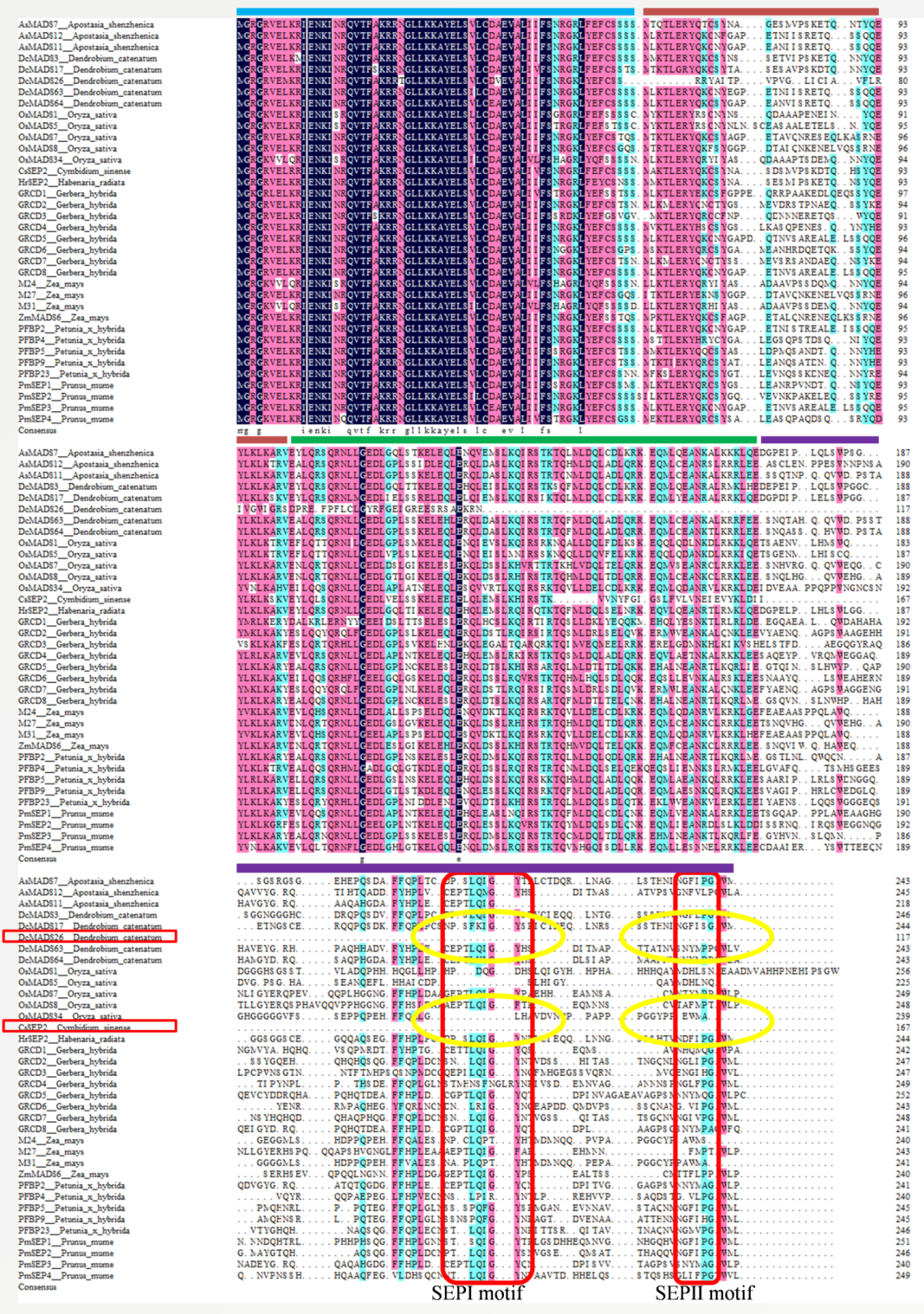


**Supplementary Figure S2** Multiple alignments of the amino acid sequence of *CsSEP2*. The *DcMADS26* protein and *CsSEP2* protein were marked by red rectangles, which possess the MADS-domain (blue line), I-domain (orange line), and K-domain (green line). Moreover, the SEP I and SEP II motifs are indicated with red boxes. The yellow lines represent the deleted domain of SEP I and SEP II.


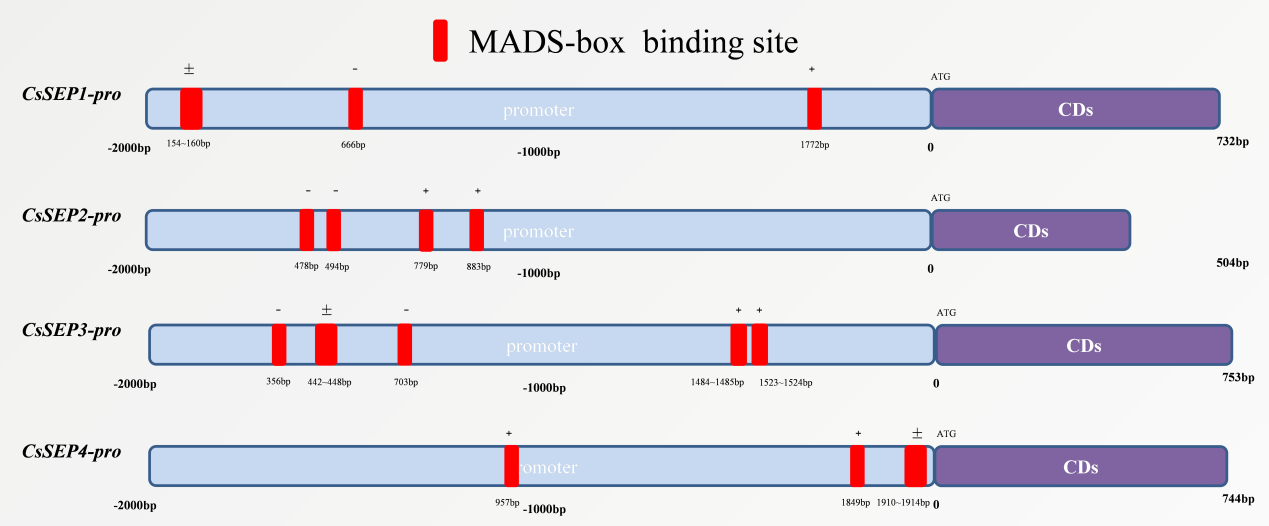


**Supplementary Figure S3** Light blue lines and short vertical bars of different colors represent promoter regions of *CsSEP1*, *CsSEP2*, *CsSEP3*, and *CsSEP4* genes and the position of putative MADS-box binding site, respectively. ＋, － and ± represent in forward, reverse complemented, and both orientations, respectively.


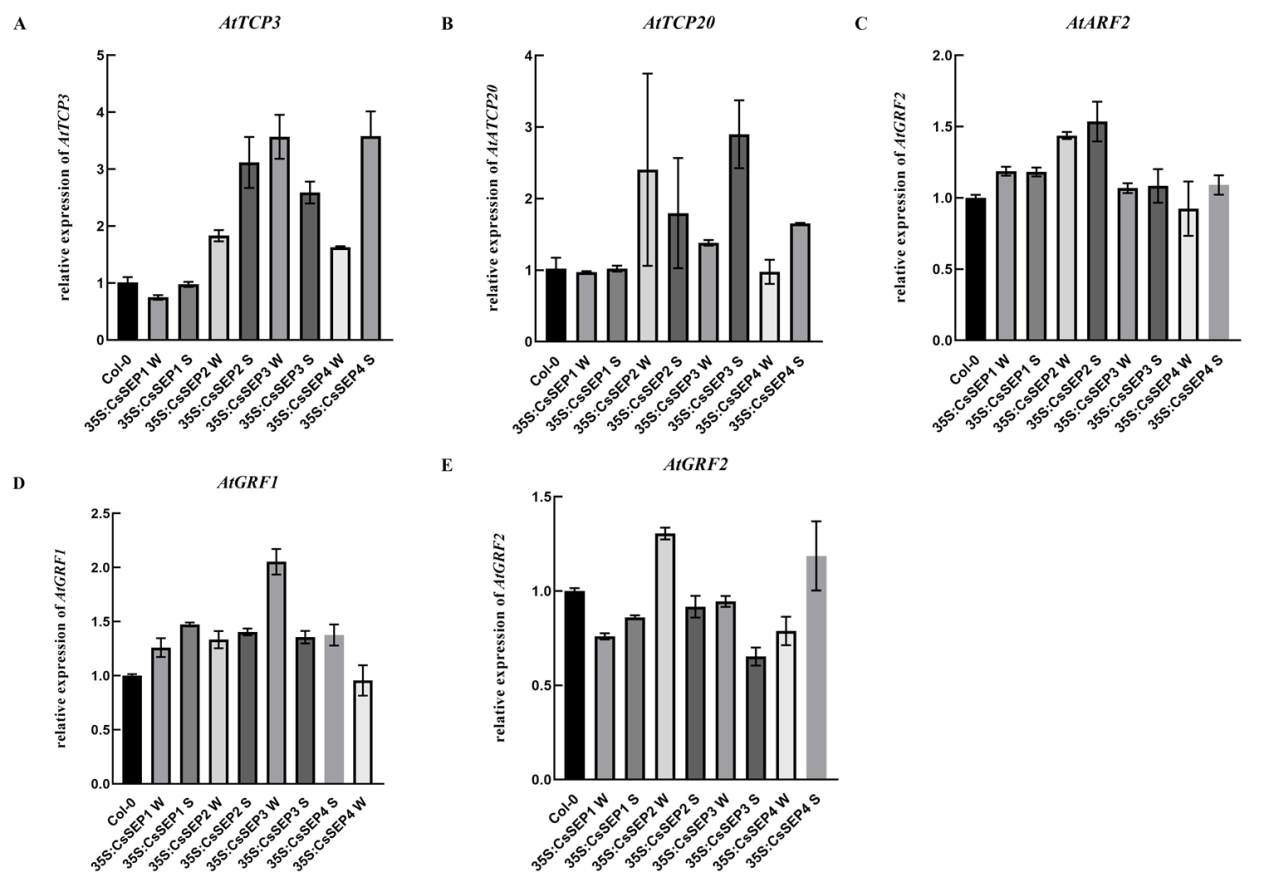


**Supplementary Figure S4** The expression of *AtTCP3*, *AtTCP20*, *AtARF2*, *AtGRF1* and *AtGRF2* endogenous genes related to leaf development were examined with qRT-PCR in Wild type Col-0, *35S:CsSEP1*, *35S:CsSEP2*, *35S:CsSEP3* and *35S:CsSEP4* transgenic *Arabidopsis*. Data are expressed as the mean of three biological replicates, with error bars indicating the SD values.

|  | ***CsSEP1*** | ***CsSEP2*** | ***CsSEP3*** | ***CsSEP4*** |
| --- | --- | --- | --- | --- |
| ***CsSEP1*** | 100.00% | 34.07% | 60.44% | 43.96% |
| ***CsSEP2*** | 34.07% | 100.00% | 39.93% | 44.32% |
| ***CsSEP3*** | 60.44% | 39.93% | 100.00% | 52.57% |
| ***CsSEP4*** | 43.96% | 44.32% | 52.57% | 100.00% |
| ***CgSEP1*** | 75.46% | NA | NA | NA |
| ***CgSEP2*** | NA | 51.65% | NA | NA |
| ***CgSEP3*** | NA | NA | 90.48% | NA |
| ***CgSEP4*** | NA | NA | NA | 100.00% |
| ***CeSEP1*** | 47.62% | NA | NA | NA |
| ***CeSEP2*** | NA | 52.01% | NA | NA |
| ***CeSEP3*** | NA | NA | 67.03% | NA |
| ***CeSEP4*** | NA | NA | NA | 100.00% |
| ***PeSEP1*** | 71.43% | NA | NA | NA |
| ***PeSEP2*** | NA | 47.25% | NA | NA |
| ***PeSEP3*** | NA | NA | 76.56% | NA |
| ***PeSEP4*** | NA | NA | NA | 86.23% |

**Supplementary Table S1**. The identity amino acid sequence of *SEP*-like proteins was aligned using DNAMAN

| Species | Gene | GenBank ID |
| --- | --- | --- |
| *Paphiopedilum henryanum* | *PhSEP1* | *MN274961* |
| *Paphiopedilum henryanum* | *PhSEP2* | *MN274962* |
| *Paphiopedilum henryanum* | *PhSEP3* | *MN809620* |
|  |  |  |
| *Petunia x hybrida* | *PMADS12* | *AY370527.1* |
| *Petunia x hybrida* | *PFBP23* | *AF335241.1* |
| *Petunia x hybrida* | *PFBP9* | *AF335236.1* |
| *Petunia x hybrida* | *PFBP5* | *AF335235.1* |
| *Petunia x hybrida* | *PFBP2* | *M91666.1* |
| *Petunia x hybrida* | *PFBP4* | *AF335234.1* |
| *Petunia x hybrida* | *PFBP29* | *AF335245.1* |
| *Petunia x hybrida* | *PFBP26* | *AF176783.1* |
|  |  |  |
| *Phalaenopsis equestris* | *PeSEP2* | *KF673858.1* |
| *Phalaenopsis equestris* | *PeSEP4* | *KF673860.1* |
| *Phalaenopsis equestris* | *PeSEP3* | *KF673859.1* |
| *Phalaenopsis equestris* | *PeSEP1* | *KF673857.1* |
|  |  |  |
| *Zea mays* | *M24* | *AJ430638.1* |
| *Zea mays* | *M31* | *AJ430640.1* |
| *Zea mays* | *M27* | *AJ430694.1* |
| *Zea mays* | *ZmMADS6* | *NM_001111683.1* |
|  |  |  |
| *Prunus mume* | *PmSEP1* | *Pm007034* |
| *Prunus mume* | *PmSEP2* | *Pm015394* |
| *Prunus mume* | *PmSEP3* | *Pm025027* |
| *Prunus mume* | *PmSEP4* | *Pm030595* |
|  |  |  |
| *Gerbera hybrida* | *GRCD1* | *AJ400623.1* |
| *Gerbera hybrida* | *GRCD2* | *AJ784156.1* |
| *Gerbera hybrida* | *GRCD3* | *AJ784157.1* |
| *Gerbera hybrida* | *GRCD4* | *FN297860.1* |
| *Gerbera hybrida* | *GRCD5* | *FN297861.1* |
| *Gerbera hybrida* | *GRCD6* | *KY708887.1* |
| *Gerbera hybrida* | *GRCD7* | *KY708888.1* |
| *Gerbera hybrida* | *GRCD8* | *KY708889.1* |
|  |  |  |
| *Habenaria radiata* | *HrSEP1* | *LC369633.1* |
| *Habenaria radiata* | *HrSEP2* | *LC369634.1* |
|  |  |  |
| *Cymbidium goeringii* | *CgSEP2* | *KX347447.1* |
| *Cymbidium goeringii* | *CgSEP3* | *MF462084.1* |
| *Cymbidium goeringii* | *CgSEP1* | *KF924272* |
| *Cymbidium goeringii* | *CgSEP4* | *APY18454.1* |
|  |  |  |
| *Cymbidium sinense* | *CsSEP1* | *Mol011084* |
| *Cymbidium sinense* | *CsSEP2* | *Mol017766* |
| *Cymbidium sinense* | *CsSEP3* | *Mol016808* |
| *Cymbidium sinense* | *CsSEP4* | *Mol018027* |
|  |  |  |
| *Arabidopsis thaliana* | *AtAP1* | *AY087956.1* |
| *Arabidopsis thaliana* | *AtAGL6* | *AT2G45650.1* |
| *Arabidopsis thaliana* | *AtAGL13* | *AT3G61120.1* |
|  |  |  |
| *Dendrobium chrysotoxum* | *Dch111658* | *Maker111658* |
| *Dendrobium chrysotoxum* | *Dch111667* | *Maker111667* |
| *Dendrobium chrysotoxum* | *Dch111954* | *Maker111954* |
| *Dendrobium chrysotoxum* | *Dch112050* | *Maker112050* |
|  |  |  |
| *Apostasia shenzhenica* | *AsMADS7* | *Ash004262* |
| *Apostasia shenzhenica* | *AsMADS12* | *Ash006741* |
| *Apostasia shenzhenica* | *AsMADS11* | *Ash018282* |
|  |  |  |
| *Cymbidium ensifolium* | *CeSEP2* | *JL009494* |
| *Cymbidium ensifolium* | *CeSEP4* | *JL018852* |
| *Cymbidium ensifolium* | *CeSEP1* | *JL009870* |
| *Cymbidium ensifolium* | *CeSEP3* | *JL004042* |
|  |  |  |
| *Dendrobium catenatum* | *DcMADS64* | *Dca016730* |
| *Dendrobium catenatum* | *DcMADS26* | *Dca011228* |
| *Dendrobium catenatum* | *DcMADS63* | *Dca018065* |
| *Dendrobium catenatum* | *DcMADS17* | *Dca003023* |
| *Dendrobium catenatum* | *DcMADS3* | *Dca021261* |

**Supplementary Table S2** The data set for *SEP*-like genes used for alignment and phylogenetic analyses in this study.

| Primer name | Sequence |
| --- | --- |
| *CsSEP1*-RT-F | **ATGGGAAGAGGGAGAGTGGAA** |
| *CsSEP1*-RT-R | **TGCAAGCCAGCCTGGTGG** |
| *CsSEP2*-RT-F | **ATGGGGAGGGGGAGAGTGG** |
| *CsSEP2*-RT-R | **GATTATGTCTAGTTTGTAAACTTCAATTTCG** |
| *CsSEP3*-RT-F | **ATGGGAAGGGGAAGAGTAGAGC** |
| *CsSEP3*-RT-R | **CTCATAGGAACCAGAAATTTGTCCA** |
| *CsSEP4*-RT-F | **ATGGGAAGGGGAAGAGTGGA** |
| *CsSEP4*-RT-R | **CATCCATCCTGGAATGAAGCC** |
| *CsSEP1*-qRT-F | **TCTGTCCTATGCGATGCT** |
| *CsSEP1*-qRT-R | **ACTCCTGCTGACTACTCTG** |
| *CsSEP2*-qRT-F | **CAAGGATACACAGCACAGT** |
| *CsSEP2*-qRT-R | **AAGAGAACCAATTCCGAAGT** |
| *CsSEP3*-qRT-F | **ACTAGCCAGACGAATACTAAC** |
| *CsSEP3*-qRT-R | **GTCCAAGCCAACCAGATG** |
| *CsSEP4*-qRT-F | **GCTCTCATCATCTTCTCCAA** |
| *CsSEP4*-qRT-R | **ATAACTGTTCTGCGTCTCC** |
| *β-actin*-qRT-F | **caatgagcttcgtgttgccc** |
| *β-actin*-qRT-R | **gatacgaaccagttgtgcgg** |
| *CsSEP1*-PAN580-F | **ggacagcccagatcaactagtATGGGAAGAGGGAGAGTGGAA** |
| *CsSEP1*-PAN580-R | **gcccttgctcaccatggatccTGCAAGCCAGCCTGGTGG** |
| *CsSEP2*-PAN580-F | **ggacagcccagatcaactagtATGGGGAGGGGGAGAGTGG** |
| *CsSEP2*-PAN580-R | **gcccttgctcaccatggatccGATTATGTCTAGTTTGTAAACTTCAATTTCG** |
| *CsSEP3*-PAN580-F | **ggacagcccagatcaactagtATGGGAAGGGGAAGAGTAGAGC** |
| *CsSEP3*-PAN580-R | **gcccttgctcaccatggatccCTCATAGGAACCAGAAATTTGTCCA** |
| *CsSEP4*-PAN580-F | **ggacagcccagatcaactagtATGGGAAGGGGAAGAGTGGA** |
| *CsSEP4*-PAN580-R | **gcccttgctcaccatggatccCATCCATCCTGGAATGAAGCC** |
| *CsSEP1*-pOCA30-F | **ctctcgagctttcgcgagctcATGGGAAGAGGGAGAGTGGAA** |
| *CsSEP1*-pOCA30-R | **cttgcatgcctgcaggtcgacTCATGCAAGCCAGCCTGG** |
| *CsSEP2*-pOCA30-F | **ctctcgagctttcgcgagctcATGGGGAGGGGGAGAGTGG** |
| *CsSEP2*-pOCA30-R | **cttgcatgcctgcaggtcgacCTAGATTATGTCTAGTTTGTAAACTTCAATTTC** |
| *CsSEP3*-pOCA30-F | **ctctcgagctttcgcgagctcATGGGAAGGGGAAGAGTAGAGC** |
| *CsSEP3*-pOCA30-R | **cttgcatgcctgcaggtcgacCTACTCATAGGAACCAGAAATTTGTCC** |
| *CsSEP4*-pOCA30-F | **ctctcgagctttcgcgagctcATGGGAAGGGGAAGAGTGGA** |
| *CsSEP4*-pOCA30-R | **cttgcatgcctgcaggtcgacTCACATCCATCCTGGAATGAAGC** |
| *CsFT*-qRT-F |  |
| *CsFT*-qRT-R |  |
| *CsSOC1*-qRT-F |  |
| *CsSOC1*-qRT-R |  |
| *CsLFY*-qRT-F |  |
| *CsLFY*-qRT-R |  |

**Supplementary Table S3** Primers used in this study.
